# Prediction of Adherence to an Online Wellness Program for People with Mobility Limitations: A Machine Learning Approach

**DOI:** 10.3390/healthcare14060781

**Published:** 2026-03-19

**Authors:** Salma Aly, Hui-Ju Young, James H. Rimmer, Tapan Mehta

**Affiliations:** 1 Department of Family and Community Medicine, Heersink School of Medicine, University of Alabama at Birmingham, Birmingham, AL 35205, USA; tapan@uab.edu; 2 Department of Health Services Administration, School of Health Professions, University of Alabama at Birmingham, Birmingham, AL 35294, USA; hjyoung@uab.edu (H.-J.Y.); jrimmer@uab.edu (J.H.R.); 3 Health Promotion and Rehabilitation Sciences, Lakeshore Foundation, University of Alabama at Birmingham, Birmingham, AL 35209, USA; 4 Center for Engagement in Disability Health and Rehabilitation Sciences (CEDHARS), University of Alabama at Birmingham, Birmingham, AL 35209, USA; 5 National Center on Health, Physical Activity and Disability (NCHPAD), University of Alabama at Birmingham, Birmingham, AL 35209, USA

**Keywords:** adherence, machine learning, telewellness, mobility limitations, digital health, MENTOR program, predictive modeling, health disparities

## Abstract

**Background/Objectives:** People with mobility limitations face disproportionately high rates of chronic health conditions and demonstrate lower adherence to wellness interventions. Digital programs such as MENTOR offer accessible alternatives but often face high rates of attrition. This study applied machine learning (ML) methods to predict adherence to the eight-week MENTOR telewellness program and identify key predictors of participant attendance. **Methods:** Data were drawn from 1218 adults enrolled in MENTOR (2023–2024). Adherence was defined as the percentage of 40 sessions attended. Baseline demographic, socioeconomic, psychosocial, mindfulness, resilience, health status, and physical activity variables were included as predictors. Following preprocessing and imputation, 13 ML regression models were trained using an 80/20 train–test split. The best-performing model was identified using mean absolute error (MAE), followed by feature selection and SHAP interpretability analyses. Pairwise synergy analysis quantified interactions between top predictors. **Results:** Model performance was modest overall. Bayesian ridge regression achieved the best performance (MAE 20.98; RMSE 25.26; R^2^ = 0.12). SHAP analyses revealed that education, race, emotional support, Area Deprivation Index, household size, mindfulness, life satisfaction, and disability onset were the strongest predictors of adherence. Higher emotional support, mindfulness, and life satisfaction were associated with greater adherence, while socioeconomic disadvantage predicted lower adherence. Synergy analyses showed the strongest predictive interactions between low education and psychosocial resources (emotional support and life satisfaction). **Conclusions:** Baseline characteristics alone modestly predicted adherence to a digital wellness program. However, psychosocial and socioeconomic factors emerged as meaningful predictors, underscoring the need for personalized support strategies to reduce dropout among participants with mobility limitations.

## 1. Introduction

Individuals with mobility limitations experience disproportionately high rates of chronic health conditions and secondary complications [1]. For example, adults with physical disabilities have a significantly higher prevalence of obesity, type 2 diabetes, cardiovascular disease, and other chronic illnesses compared to the general population [2]. Structured health promotion and wellness programs can help prevent disease and improve the quality of life for these individuals. However, the effectiveness of any program depends largely on participant adherence to the prescribed regimen. Adherence is a pivotal determinant of intervention success: poor adherence undermines clinical effectiveness, leading to disease progression, complications, and increased healthcare costs, whereas good adherence supports better disease control and improved quality of life [3]. In the context of lifestyle and digital interventions, nonadherence is particularly problematic. Health intervention trials delivered via digital platforms frequently report high attrition, with dropout rates as high as 72–83% [3]; this pattern reflects the so-called “law of attrition,” [4] in which a substantial proportion of participants discontinue use of the intervention or withdraw from the study before completion.

Adherence to health promotion programs tends to be lower in people with mobility limitations when compared to the general population [2,5,6]. Low adherence limits the programs’ impact, as those who could benefit most often attend least. In general, sustaining participation in wellness programs is challenging for people with disabilities [7]. Barriers to adherence are multifactorial. On the participant level, motivational and psychosocial factors often erode engagement. Users commonly cite low motivation and lack of engagement as reasons for dropping out [8]. Practical constraints such as time limitations and repetitive or tedious content also discourage continued participation [8]. Environmental and social factors further complicate adherence. People with disabilities may encounter inaccessible facilities or transportation barriers, and those in rural or low-income settings may lack local resources [2,9]. Moreover, inadequate digital infrastructure, such as limited device availability or unreliable internet, remains a fundamental barrier to online participation [10,11]. Technological and usability issues can also make adherence a challenge.

Digital wellness programs introduce both advantages and new barriers to adherence. Delivering interventions online can mitigate certain barriers—for example, participants can join classes from home to avoid transportation or scheduling conflicts [2]. However, research shows that many eHealth interventions are not as engaging as in-person programs, which can lead to lower attendance and higher attrition [3,12]. Without in-person accountability, users often disengage over time. One review notes that even interventions showing promising short-term outcomes often see long-term effectiveness diminish due to poor retention [13,14]. Thus, while digital delivery enhances accessibility, it is not a comprehensive solution. Sustained adherence requires proactive strategies that address motivational, social, and technical barriers.

Given the central role of adherence, researchers have increasingly applied machine learning (ML) techniques to predict and prevent dropouts. By analyzing user behavior data, ML models can identify individuals at risk for non-adherence and enable timely, personalized interventions. A recent systematic review (2023) found growing application of supervised ML models to predict adherence in behavior-change support systems, with many achieving good classification accuracy [15]. For instance, Pedersen et al. (2019) built a random forest model that correctly identified 89% of dropouts from a diabetes eHealth lifestyle program [3]. Accurate early prediction of non-adherence allows programs to deliver targeted re-engagement efforts when participants begin to disengage. ML-driven prediction can “provide users with more personalized, tailored, and timely suggestions” to reinforce adherence [15].

The National Center on Health, Physical Activity and Disability (NCHPAD), based at the University of Alabama at Birmingham, offers an eight-week comprehensive online wellness program called MENTOR (Mindfulness, Exercise, and Nutrition to Optimize Resilience). By addressing both physical and mental health through a synchronous, “high touch” virtual format, MENTOR aims to build resilience and prevent secondary conditions in people with mobility limitations. Adherence to the program was operationally defined as the proportion of the 40 program sessions that each participant attended.

To date, the mean attendance has been only about 32% of sessions, suggesting that most participants are not completing the full “dose” of this comprehensive wellness program and may therefore experience limited benefits. However, little is known about which participants remain engaged and why.

Thus, the purpose of the present study was to develop an ML model to forecast individual adherence to the eight-week MENTOR program and identify the key predictors of adherence. By applying data-driven prediction, we aimed to pinpoint at-risk participants early and understand the factors that most strongly influence adherence. Ultimately, our goal was to enhance MENTOR’s impact by guiding tailored support strategies for individuals with mobility limitations, thereby improving health outcomes in this population.

## 2. Methods

### 2.1. Data Source and Participants

The MENTOR program is an eight-week telewellness intervention designed for adults with mobility limitations. Delivered entirely online via the Healthie (Healthie Inc., San Francisco, CA, USA) platform, the program consists of 40 live sessions—five per week—covering adapted exercises, nutrition education, guided mindfulness, and health coaching. Participants receive at-home fitness equipment and access to the Healthie portal, which facilitates goal tracking, home practice, and interaction with wellness coaches and peers. The program emphasizes resilience building by integrating physical, mental, and emotional wellness strategies into each weekly module, aiming to empower participants to better manage their health and enhance their overall quality of life [16]. Data included participants registered in NCHPAD’s longitudinal registry, NCHPAD Connect, and enrolled into the MENTOR program from January 2023 to July 2024.

### 2.2. Study Variables

Our outcome variable is program adherence, measured as the percentage of sessions attended out of 40, resulting in a continuous measure of participant engagement for use as the predictive outcome. Attendance data is captured via the Healthie platform. We included a range of independent variables in our predictive models, beginning with baseline, self-reported questionnaire data collected when participants first enrolled in NCHPAD Connect. These variables encompass demographic characteristics such as age, gender, marital status, residence location, and the national Area Deprivation Index (ADI) [17] to capture socioeconomic context. The disability onset was categorized into early and late. Early-onset disability refers to conditions that manifest in childhood or adolescence (developmental period) and persist into adulthood [18], e.g., cerebral palsy, spina bifida, and congenital genetic disorders. Late-onset disability denotes impairments that emerge in adulthood, often due to acquired conditions, i.e., spinal cord injury, multiple sclerosis, Parkinson’s, Guillain–Barré syndrome, or fibromyalgia [19].

Access to healthcare was assessed using multiple indicators, including health insurance coverage, receipt of supplemental benefits (e.g., Supplemental Security Income, Supplemental Nutrition Assistance programs), engagement in routine healthcare services such as dental and eye examinations, participation in preventive care (e.g., cancer screenings and vaccinations), access to specialty care through referrals, and indicators of chronic condition management including the presence of comorbidities. Additionally, we incorporated other psychosocial, resilience, mindfulness, and wellness measures as summarized in Table 1.

All measures were administered via a secure online platform (Research Electronic Data Capture [REDCap; Vanderbilt University, Nashville, TN, USA]). For the present analysis predicting adherence, we used the baseline (pre-program) scores from these wellness measures as independent variables, which, together, provided a comprehensive profile of each participant. This allowed our ML model to identify key predictors of adherence, enabling targeted intervention strategies to support continued engagement in the MENTOR program.

### 2.3. Statistical Analysis

Data cleaning and descriptive analyses were conducted using R software (version 4.4.2; R Foundation for Statistical Computing, Vienna, Austria) [26]. Descriptive statistics for categorical variables are presented as frequencies and percentages, while continuous variables are reported as means with standard deviations (SD).

### 2.4. Data Preprocessing

The dataset was randomly divided into 80% for model development (training set) and 20% for testing. The proportion of missing data across variables ranged from approximately 0.1% to 17%, with the highest missingness observed for the PROMIS Global Physical Health and PROMIS Global Mental Health scores, and the lowest missingness observed for gender, the PROMIS Emotional Support score, and the NCHPAD wellness assessment score. Missing data for continuous variables were imputed using the K-Nearest Neighbor (KNN) imputation technique [27]. For categorical variables, the Frequency Category (mode) Imputation technique was applied, followed by one-hot encoding. All preprocessing steps, including imputation and encoding, were performed using the training data only, and the same transformations were subsequently applied to the test data to prevent potential information leakage.

### 2.5. Feature Selection and Dimensionality Reduction

The model was selected based on the resulting lowest mean absolute error (MAE) [28], and dimensionality reduction was performed using recursive feature elimination (RFE) [29] and three regularization techniques [30] Least Absolute Shrinkage and Selection Operation (LASSO), ridge, and elastic net regression, with the lowest MAE resulting from elastic net regression. Feature selection procedures were conducted using the training data only to avoid information leakage.

**Figure 1:**
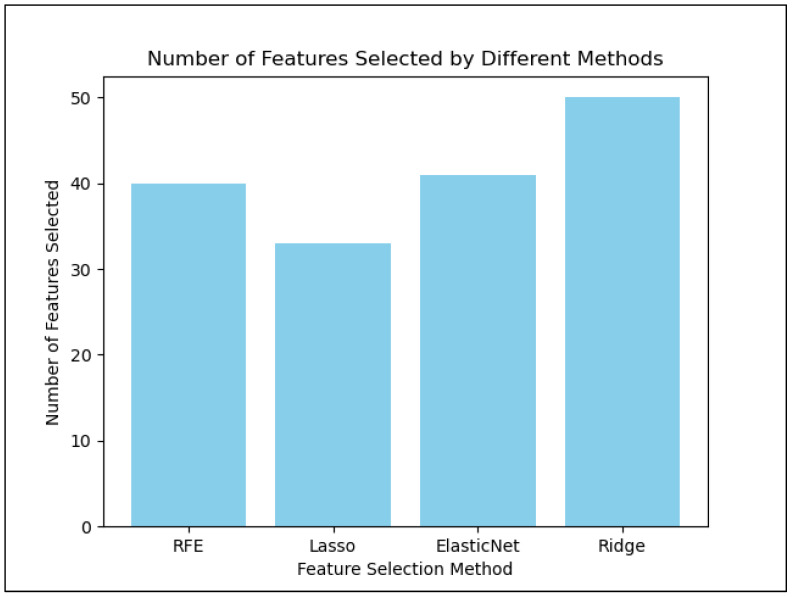
Features selection by different dimensionality reduction techniques. Abbreviations: RFE: recursive feature elimination.

Feature selection was implemented to reduce redundancy among predictors, mitigate potential multicollinearity, and improve model interpretability and generalization performance. Although the reduction in predictors was modest (50 to 42 variables), the procedure helped retain the most informative variables while minimizing noise from less informative predictors. Figure 1 illustrates the number of variables selected by each dimensionality reduction technique. The variables were reduced from 50 to 42, and the features selected are shown in Table 2.

### 2.6. Regression Models

After preprocessing, 13 ML regression algorithms were employed on the training dataset. These algorithms encompassed decision tree (DT), support vector machine (SVM), Naïve Bayes (NB), linear, random forest (RF), adaptive boosting (AdaBoost), eXtreme Gradient Boosting (XGBoost), CatBoost, gradient boosting, Bayesian ridge, Huber, Extra Trees, and Light Gradient-Boosting Machine (LightGBM) regression models. Hyperparameters for candidate models were optimized using grid search combined with 5-fold cross-validation when applicable. Some models, such as linear regression and Bayesian ridge regressions, estimate their regularization parameters internally during model fitting rather than requiring extensive hyperparameter tuning. Preprocessing and subsequent analysis were conducted utilizing Python (version 3.10.5; Python Software Foundation, Wilmington, DE, USA) [31].

### 2.7. Model Validation

To evaluate model performance and reduce potential overfitting, 5-fold cross-validation (CV) was applied to the training dataset [32]. This dataset was randomly partitioned into five equal subsets (folds). In each iteration, four folds were used for model training and the remaining fold for validation, and the process was repeated until each fold served once as the validation set. The performance metrics obtained from the five folds were averaged to provide a robust estimate of model generalization performance.

### 2.8. Evaluation Criteria

The best-performing model was selected based on the lowest mean absolute error (MAE) obtained from the 5-fold cross-validation procedure. MAE was chosen because it provides a direct and interpretable measure of the average magnitude of prediction error and is less sensitive to extreme values than squared-error metrics [33]. Additional performance measures included root mean square error (RMSE) and the coefficient of determination (R^2^). For each metric, results were calculated within each fold of the cross-validation procedure and summarized as the mean performance across the five folds with corresponding 95% confidence intervals.

### 2.9. Feature Importance Stability

Feature importance was computed for the final selected model within each fold of the 5-fold CV procedure. To assess the stability of feature importance, the coefficients were calculated separately in each fold and then averaged across folds, and their variability was summarized using the standard deviation [34]. Feature importance stability reflects the robustness of feature rankings when the training data vary across different samples [35].

### 2.10. Model Interpretation

SHapley Additive explanations (SHAP) is the average marginal contribution of a variable considering all possible combinations [36]. These were calculated to identify the highest predictors of adherence and their effect on model outcome.

### 2.11. Synergy (Pairwise Interaction) Analysis

To quantify pairwise interactions among the top 10 predictors, we conducted a performance-based synergy analysis on the held-out test set. For each predictor xi, a baseline model was fit using xi alone, and for each pair (xi, xj), a two-predictor model was fit. We then computed MAE for each single- and two-predictor model. Our primary synergy metric was:

ΔMAE_ij_ = 1/2(MAE_i_ + MAE_j_) − MAE_ij_

so that positive values indicate that the pair reduces error more than expected from the average of the single-predictor models, consistent with performance-based interaction concepts in the literature [37]. For visualization, non-positive values were masked, and ΔMAE was displayed as a heat map. This approach is closely related to Oh’s prediction-performance interaction measure, which compares the joint error change under dual perturbation to the sum of individual changes. We emphasize that these quantities reflect predictive interactions rather than causal effect modification.

### 2.12. Code Availability

Code was written in Python programming language using scikit-learn libraries (scikit-learn developers, global open-source project) and is available upon request.

## 3. Results

Table 3 summarizes the baseline characteristics of the participants, with a mean age of 55.5 years, two-thirds identifying as female, and the majority identifying as White, non-Hispanic, and residing in urban areas. The median percentage of sessions attended was 30% (IQR: 5–55).

As shown in Table 4, model performance was modest overall. The reported metrics represent the mean performance across the five CV. Bayesian ridge regression exhibited the best reported overall fit (MAE 20.68; RMSE 25.54; R^2^ = 0.12). CatBoost (MAE = 21.27; RMSE = 26.74; R^2^ = 0.08) and ordinary linear regression (MAE = 22.24; RMSE = 26.45; R^2^ = 0.11) demonstrated similar predictive performance, with slightly higher prediction errors than Bayesian ridge regression. Several tree-based and neighborhood methods performed worse: the decision tree showed the poorest performance (MAE = 28.53; RMSE = 36.35; R^2^ = −0.70), and k-nearest neighbors also performed relatively poorly (MAE = 25.95; RMSE = 30.77; R^2^ = −0.22).

Feature importance analysis for the Bayesian Ridge regression is presented in Table 5. Predictors were ranked according to the absolute magnitude of their mean coefficients obtained across the 5-fold CV procedure. The most influential predictors included high school education, number of households, and the Emotional Support Score. The relatively small standard deviations across folds indicate that the importance rankings were stable despite variations in the training data.

**Figure 2:**
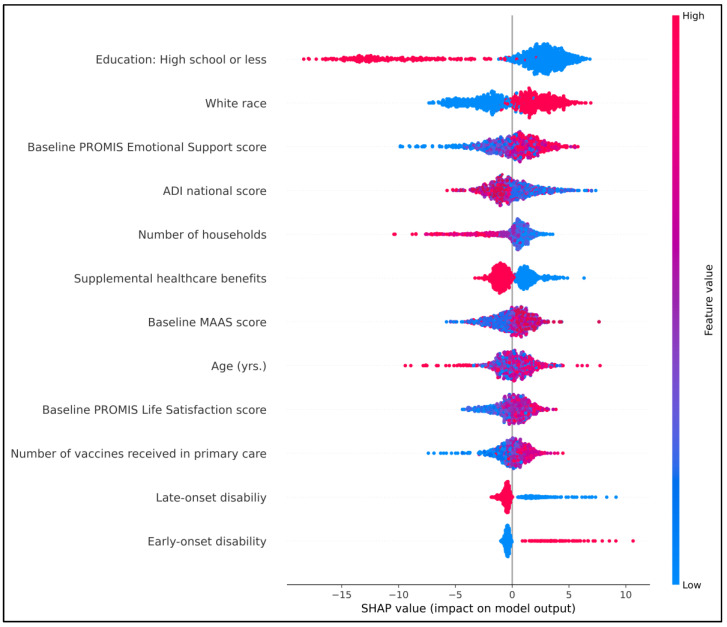
SHAP summary (bee swarm) plot showing variables’ importance for percentage adherence prediction model Abbreviations: PROMIS: Patient-Reported Outcomes Measurement Information System; ADI: Area Deprivation Index; MAAS: Mindful Attention Awareness Scale.

The SHAP summary plot presents the top 12 features ranked by their mean absolute SHAP values, representing their average contribution to the model’s predictions h (Figure 2). Features are ordered in descending importance, and the plot illustrates how variation across each feature’s values influences the predicted percentage adherence [36]. Color denotes feature value (red = high, blue = low), with points to the right of zero indicating a positive contribution to predicted adherence and points to the left indicating a negative contribution.

In this model, socioeconomic and psychosocial features dominated the ranking: educational attainment (“high school or less”), race (White), baseline PROMIS Emotional Support, ADI, household size, and receipt of supplemental benefits appeared among the most important predictors. Baseline mindfulness (MAAS) and life satisfaction (PROMIS Life Satisfaction) measures also contributed substantially, as did age and indicators of disability onset (early/late). Inspection of point distributions indicates that higher baseline emotional support, higher mindfulness, and greater life satisfaction tended to push predictions toward higher adherence (red points more often on the right), whereas markers of greater socioeconomic disadvantage (higher ADI, lower education, and indicators of supplemental benefits) and some disability-related indicators tended to push predictions toward lower adherence (red points more often on the left). Notably, White race, a higher number of vaccines received in primary care, and larger household size are associated with higher predicted adherence, as higher values of these features correspond to SHAP points to the right, indicating consistent positive contributions to the model’s adherence predictions.

**Figure 3:**
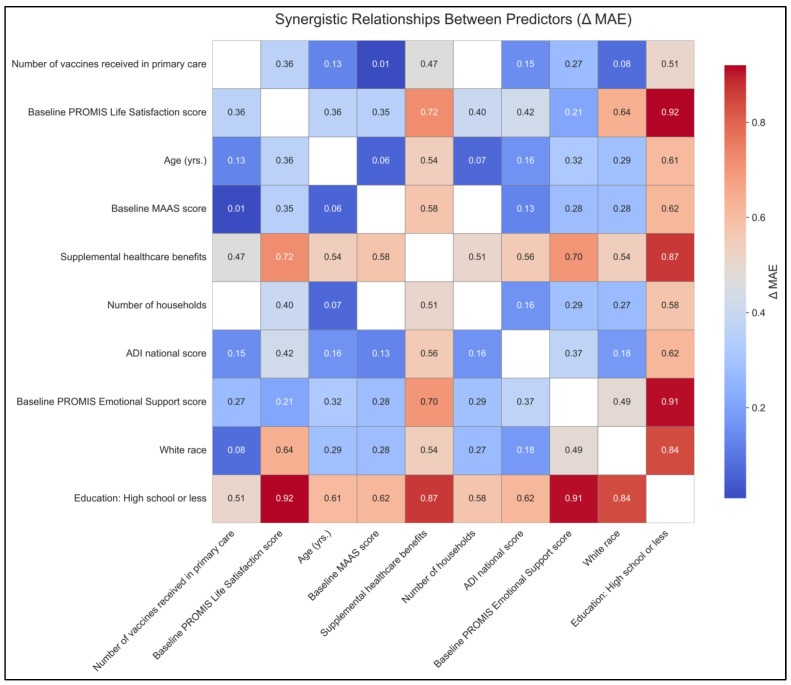
Synergy heatmap of feature pairs based on Δ MAE for predicting percentage adherence in the MENTOR program Abbreviations: MAE: mean absolute error; ADI: Area Deprivation Index; MAAS: Mindful Attention Awareness Scale. ΔMAE: the average of two individual feature models’ mean absolute errors minus the mean absolute error from a model trained using both features together.

The synergy matrix summarizes pairwise predictive interactions among the top baseline predictors of adherence. The largest synergies involved education (high school or less) combined with psychosocial resources, with PROMIS Life Satisfaction (ΔMAE ≈ 0.92) and PROMIS Emotional Support (ΔMAE ≈ 0.91), followed by receipt of supplemental benefits (ΔMAE ≈ 0.87). Additional moderate synergies were observed for life satisfaction with supplemental benefits (ΔMAE ≈ 0.72) and White race with life satisfaction (ΔMAE ≈ 0.64). Smaller or near-zero synergies were seen for pairs involving ADI and number of households. Collectively, these patterns suggest that socioeconomic context (education, benefits) and perceived social/emotional resources interact to enhance predictive accuracy for program adherence, beyond their individual effects (Figure 3).

## 4. Discussion

Our ML model demonstrated modest predictive performance (MAE ≈ 21), consistent with findings in the broader literature on adherence to digital interventions [38,39,40,41]. A recent systematic review, for example, reported that sociodemographic and health predictors of eHealth engagement often show mixed and inconsistent associations [41]. Adherence is often influenced by unmeasured factors such as motivation, life events, and intervention usability, resulting in low explained variance across studies. Thus, while an R^2^ of 0.12 indicates considerable unexplained variability, it aligns with prior studies demonstrating only weak to moderate success in predicting adherence from baseline variables [41,42]. Given the inherent complexity of adherence behavior, models relying solely on baseline characteristics are unlikely to achieve high predictive accuracy. In this context, the primary value of the present model lies in risk stratification—identifying individuals who may be at increased risk of low adherence—rather than precise individual-level prediction. Several psychosocial predictors emerged as important in our analyses. Although the overall predictive performance of our ML model was modest, as reflected in limited accuracy metrics, examination of feature contributions using SHAP provided valuable insights into potential determinants of adherence, consistent with contemporary interpretability practices in ML [43,44,45]. In our study, emotional support from family or peers was positively associated with adherence, aligning with prior research demonstrating the efficacy of social facilitation in enhancing engagement in digital health interventions. For instance, reviews of mobile health interventions have shown that individuals with higher perceived social support are more likely to stay engaged [46]. Mindfulness also appeared to be a relevant factor: participants with higher mindfulness scores tended to engage more consistently. Related work in mindfulness-based programs has shown that baseline characteristics such as conscientiousness and openness predict better out-of-class practice adherence [47]. Analogously, greater mindfulness and self-regulatory capacity may facilitate sustained adherence to program protocols. Finally, socioeconomic disadvantage emerged as a risk factor for lower adherence, with lower income and education associated with higher attrition. A nationwide survey of individuals with chronic disease reported significantly lower completion of digital assessments among lower-income participants [48], and a systematic review of telerehabilitation programs identified lower education as a predictor of non-participation [49]. Collectively, these findings suggest that participants facing socioeconomic barriers may benefit from additional support such as tailored coaching or resource assistance to promote sustained engagement.

Because several predictors in our model included sensitive sociodemographic variables such as race, education, and income, their interpretation requires careful consideration. ML models trained on observational health data can sometimes reflect existing social or structural inequalities present in the underlying datasets. Previous studies have highlighted that algorithms may unintentionally reproduce disparities if such variables are interpreted without appropriate context [50,51]. For example, a widely cited study demonstrated that a healthcare algorithm used to identify patients for additional care underestimated the needs of Black patients because it relied on healthcare spending as a proxy for health need, which systematically underestimated illness burden among populations with historically lower access to care [52]. In this study, these variables were included primarily to capture potential structural barriers influencing engagement with digital interventions rather than to imply intrinsic differences between individuals.

Our synergy analysis illuminated important interactions among risk factors. For example, individuals with both low education and low emotional support exhibited particularly poor adherence, which is consistent with prior evidence that a “double burden” of disadvantage amplifies dropout risk. People with lower education often have limited digital literacy and may struggle to engage without a supportive network [49]. Conversely, strong emotional support can buffer educational gaps. These interactions have clear practical implications for intervention design. For participants with lower education, proactive strategies such as simplified instructions or peer mentoring may be beneficial, while for those lacking emotional support, integrating group chats or other social features may help foster connectedness. Digital health research suggests that social support components such as discussion forums or coach check-ins can increase adherence, but their effectiveness may depend on alignment with user needs [46]. Overall, our findings highlight the value of addressing multiple, co-occurring risk factors (e.g., education, income, social support) to more accurately stratify adherence risk and inform personalized engagement strategies.

The use of ML offered both opportunities and challenges. On the positive side, ML algorithms can capture complex, non-linear relationships and integrate many features beyond the capacity of traditional regression approaches [42]. For example, studies of online depression interventions have shown that incorporating early engagement data, such as first-week logins and time spent on the platform, substantially improved predictive performance. Models including these variables achieved high accuracy, whereas those relying only on demographic characteristics performed poorly [42]. Similarly, our approach could identify subtle predictors of engagement and automatically flag users at elevated risk of dropout. This flexibility is particularly valuable in digital health, where adherence behaviors are dynamic and highly context dependent.

However, ML also has limitations in this domain. Even advanced models often explain only a modest proportion of variance in adherence, highlighting the influence of unobserved behavioral and contextual factors [41]. In our study, the modest R^2^ suggests that many key determinants, such as intrinsic motivation, life stressors, competing clinical appointments, or not feeling well physically or mentally, remain unmeasured. Moreover, deploying ML models in populations with disabilities requires particular caution. Digital interventions must be accessible (e.g., compatible with screen readers or an adjustable interface) and responsive to various user needs [53]. Models trained on populations with mobility limitations may not generalize to other groups without retraining or adaptation. Overall, while ML holds considerable promise for enhancing prediction and personalization in digital wellness interventions, its outputs should be interpreted cautiously and supplemented with domain expertise and comprehensive design principles.

Our study has several limitations. First, predictive power was limited: R^2^ ≈ 0.12 means the model captured only a small portion of variance. This modest performance is inherent to behavior modeling and highlights that many adherence determinants were not captured. Second, all independent variables were self-reported, introducing potential bias. Thus, our adherence outcome (session attendance) may likewise be misremembered or inflated, which could weaken model accuracy. Third, generalizability is uncertain. Our sample was large and national, but with more representation from the Deep South. Patterns of engagement could differ in younger populations, other disability groups, or non-USA settings. Finally, our interpretations rely on SHAP and ΔMAE, which highlight associations but not causality. Thus, we cannot conclude that increasing emotional support will lead to better adherence. Future work should test whether modifying key adherence factors leads to changed behavior.

In the present study, the prediction models were intentionally designed to use baseline demographic, social, and psychosocial characteristics collected prior to the start of the program. This approach allows early identification of individuals who may be at risk of low adherence before participation begins, enabling proactive support strategies.

Nevertheless, future models should incorporate temporal usage data such as early login frequency, weekly attendance and session duration. Including these temporal features would allow more accurate tracking of engagement patterns and their trends over time. Integrating predictive algorithms into real-time monitoring systems could further enhance their impact by enabling automated alerts or coach check-ins for participants identified as high-risk of dropout. Such timely, targeted interventions would help optimize program resources and improve retention.

Model validation on independent cohorts will be essential to assess robustness and generalizability. Given the heterogeneity of disability, developing subgroup-specific models may also improve predictive performance. Finally, combining ML with behavioral theory may yield more interpretable and effective predictions. Hybrid approaches that integrate theoretical constructs such as self-efficacy or stages of change could inform the design of personalized, theory-informed interventions.

## 5. Conclusions

Our findings highlight several clinically meaningful opportunities. Most importantly, even relatively modest ML models can identify at-risk users early. As Wenger et al. note, predicting non-adherence from baseline characteristics or early engagement patterns allows proactive intervention [42]. In practice, such a system could assign dropout-risk scores to new enrollees and alert coaches to intervene (e.g., by a phone call or motivational message) before disengagement occurs. Beyond early detection, understanding which factors drive risk allows for more personalized coaching. If low social support is identified, coaches might emphasize community features or increase one-on-one check-ins. If low mindfulness or self-regulation emerges, digital content can be tailored with timely reminders to help participants stay on track. By integrating these predictive tools into wellness platforms, programs such as MENTOR can move beyond one-size-fits-all approaches toward truly adaptive support, potentially improving outcomes for people with mobility limitations.

## Figures and Tables

**Table 1:** Variables assessed and their measurement tools.

Variables	Tools
Psychosocial measures	PROMIS General Life Satisfaction scale [20] PROMIS Emotional Support short form [21]
Resilience	10-item Connor-Davidson Resilience Scale (CD-RISC-10) [22]
Mindfulness	15-item Mindful Attention Awareness Scale (MAAS) [22]
Physical activity behavior	Godin Leisure-Time Exercise Questionnaire (GLTEQ) [23]
General health status	PROMIS Global Health 10 assessment with physical and mental health subscales [24]
Wellness	16-item NCHPAD Wellness Assessment, a multidimensional instrument covering physical, mental, and emotional wellness domains [25]

Abbreviation: PROMIS: Patient-Reported Outcomes Measurement Information System; NCHPAD: National Center on Health, Physical Activity and Disability.

**Table 2:** Variables selected after using the elastic net regression dimensionality reduction technique.

Variables		
**Socio-demographic variables**		
Age		
Gender		
Race and ethnicity		
Marital status		Living with partner
		Living without partner
Rural residence (Micropolitan)		
Annual income		
Number of households		
Age at time of injury		
Employment		
Education		High school or less
		Associate or Bachelor
		Postgraduate
Area Deprivation Index national score		
**Clinical diagnosis and access to care**		
Onset of physical disability (Late vs. Early onset) **		
Presence of comorbidity		
Having healthcare screening through a primary care doctor within the past 12 months		
Number of vaccinations received		
Receiving supplemental healthcare benefits		
Having routine health checkups		
Dental examination		
Ophthalmologic examination		
**Psychosocial scores**		
PROMIS General Life Satisfaction score		
PROMIS Emotional Support score		
Connor-Davidson Resilience Scale		
Mindfulness Attention Awareness Scale		
PROMIS Global Health score	Physical	
	Mental	
NCHPAD Wellness Assessment scores	Physical	
	Mental	
	Emotional	
Level of physical activity measured by Godin Leisure-Time Exercise Questionnaire		

Excluded features: Having any type of health insurance, living in metropolitan areas. ** Early-onset disability refers to conditions that manifest in childhood or adolescence (developmental period) and persist into adulthood [18], e.g., cerebral palsy, spina bifida, and congenital genetic disorders. Late-onset disability denotes impairments that emerge in adulthood, often due to acquired conditions, i.e., spinal cord injury, multiple sclerosis, Parkinson’s, Guillain–Barré syndrome, or fibromyalgia [19].

**Table 3:** Descriptive characteristics of study participants.

Characteristics	(*n* = 1218)
Age, mean (SD) years	55.5 (14.3)
Gender (female), *n* (%)	798 (65.5%)
Marital status	
Living without partner, *n* (%)	695 (57.1%)
Living with partner, *n* (%)	479 (39.3%)
Unknown, *n* (%)	44 (3.6%)
Race	
White, *n* (%)	658 (54.0%)
Black, *n* (%)	394 (32.3%)
Other, *n* (%)	81 (6.7%)
Unknown, *n* (%)	85 (7.0%)
Ethnicity (non-Hispanic), *n* (%)	1130 (92.8%)
Residence (Urban), *n* (%)	1081 (88.8%)
Area Deprivation Index, mean (SD)	49.4 (29.3)
Educational level	
High school or less, *n* (%)	291 (23.9%)
Bachelor’s degree or similar, *n* (%)	675 (55.4%)
Postgraduate, *n* (%)	241 (19.8%)
Unknown, *n* (%)	11 (0.9%)
Employment (yes), *n* (%)	204 (16.7%)
Annual income	
Less than $50,000, *n* (%)	655 (53.8%)
$50,000–$100,000, *n* (%)	192 (15.8%)
More than $100,000, *n* (%)	113 (9.3%)
Unknown, *n* (%)	258 (21.2%)
Percentage of sessions attended, median (IQR)	30% (5%, 55%)

**Table 4:** Performance of different regression models for predicting percentage adherence to the MENTOR program using 5-fold cross-validation with hyperparameter tuning.

Regression Model	MAE (95% CI)	RMSE (95% CI)	R^2^ (95% CI)
Decision Tree	28.53 (27.72, 29.35)	36.35 (35.39, 37.31)	−0.70 (−0.77, −0.63)
KNN	25.95 (25.04, 26.86)	30.77 (29.67, 31.88)	−0.22 (−0.28, −0.15)
SVM	24.64 (23.40, 25.88)	28.19 (26.99, 29.39)	−0.02 (−0.09, 0.04)
XGBoost	24.08 (23.44, 24.72)	28.97 (28.07, 29.87)	−0.08 (−0.14, −0.02)
AdaBoost	23.95 (22.95, 24.95)	27.45 (26.56, 28.34)	0.03 (−0.01, 0.07)
Extra Trees	23.67 (22.35, 25.00)	28.30 (26.91, 29.70)	−0.03 (−0.13, 0.07)
Huber	22.89 (22.00, 23.78)	27.49 (26.32, 28.65)	0.02 (−0.08, 0.13)
LightGBM	22.80 (21.70, 23.90)	27.47 (26.57, 28.36)	0.03 (−0.02, 0.07)
Random Forest	22.65 (21.55, 23.75)	26.83 (25.79, 27.86)	0.07 (0.03, 0.12)
Gradient Boosting	22.62 (21.68, 23.56)	26.83 (26.00, 27.67)	0.07 (0.04, 0.10)
Linear Regression	22.24 (20.77, 23.70)	26.45 (25.08, 27.83)	0.11 (0.03, 0.17)
CatBoost	21.27 (20.30, 22.25)	26.74 (25.86, 27.62)	0.08 (0.03, 0.13)
Bayesian Ridge **	20.68 (19.58, 22.78)	25.54 (24.47, 26.60)	0.12 (0.07, 0.14)

Abbreviations: MAE: mean absolute error; RMSE: root mean square error; R^2^: coefficient of determination; SVM: support vector machine; XGBoost: eXtreme Gradient Boosting; KNN: k-nearest neighbors; AdaBoost: adaptive boosting; LightGBM: Light Gradient Boosting Machine; ** Best performing model is Bayesian ridge regression.

**Table 5:** Top 12 predictors ranked by mean standardized coefficient with stability across 5-fold cross-validation for the Bayesian Ridge regression model.

Feature	Mean Coefficient	SD
Education: High school or less	−3.12	0.43
Number of households	−2.16	0.10
Baseline PROMIS Emotional Support score	2.12	0.31
Early-onset disability	1.95	0.35
Supplemental healthcare benefits	−1.86	0.12
White race	1.73	0.22
Age in years	−1.72	0.40
Baseline PROMIS Life Satisfaction score	1.47	0.11
Late-onset disability	−1.35	0.19
Number of vaccines received in primary care	1.22	0.15
ADI national score	−1.15	0.31
Baseline Global Physical Health PROMIS score	1.09	0.37

Abbreviations: PROMIS: Patient-Reported Outcomes Measurement Information System; ADI: Area Deprivation Index.

## Data Availability

Deidentified data supporting the findings of this study are available from the authors upon reasonable request. Due to privacy and ethical restrictions related to participant confidentiality, the data are not publicly available.
